# Factors Influencing Prevalence, C-reactive Protein Levels, and Lymphocyte Counts in Chronic Obstructive Pulmonary Disease Patients With Metabolic Syndrome

**DOI:** 10.7759/cureus.68122

**Published:** 2024-08-29

**Authors:** Binod Mahato, Shreya Nigoskar, Lingidi Jhansi Lakshmi, Doddigarla Zephy

**Affiliations:** 1 Department of Biochemistry, Index Medical College, Hospital & Research Centre, Malwanchal University, Indore, IND; 2 Department of Biochemistry, Hi-Tech Medical College & Hospital, Rourkela, IND

**Keywords:** height, waist circumference, diastolic pressure, systolic pressure, chronic obstructive pulmonary disease, metabolic syndrome

## Abstract

Background

This research examined the relationship between C-reactive protein (CRP) levels and lymphocyte counts in individuals with chronic obstructive pulmonary disease (COPD) comparing those with metabolic syndrome (MetS) to those without.

Methodology

This cross-sectional study involved 100 consecutive COPD patients attending the outpatient wards at the Department of Medicine, Index Medical College, over 18 months. MetS was assessed using the International Diabetes Federation’s guidelines. Pulmonary function tests such as spirometry were conducted following the European Respiratory Society’s procedures, including measurements of forced expiratory volume in one second (FEV1), forced vital capacity (FVC), and the FEV1/FVC ratio. The Global Initiative for Obstructive Lung Disease criteria were employed to evaluate COPD severity using post-bronchodilator FEV1.

Results

Our results indicated no significant differences in demographics, anthropometrics, or pulmonary function tests between COPD patients with MetS and those without. Average age, height, weight, body mass index, and blood pressure readings were similar between the groups, with no significant variations (p > 0.05). However, the total white blood cell count was significantly higher in the MetS group (9,214 ± 3,161.8 cells/µL) compared to the non-MetS group (6,657.8 ± 4,218 cells/µL, p = 0.001). CRP levels were markedly elevated in 90.9% of MetS patients compared to 21.4% of non-MetS patients. Pulmonary function tests showed no significant differences in pre- and post-bronchodilator FEV1 or FEV1/FVC ratios (p > 0.05).

Conclusions

The study found that individuals with COPD and MetS have elevated levels of CRP, suggesting that this association exacerbates systemic inflammation and metabolic issues. Furthermore, the risk of MetS in COPD patients did not significantly differ between smokers and non-smokers, indicating that MetS can affect all COPD patients regardless of smoking status. Additionally, more than half of the COPD patients had hypertension, a common comorbidity that reflects the oxidative stress and inflammatory processes shared by both conditions.

## Introduction

Metabolic syndrome (MetS) is a collection of risk factors that significantly increase the likelihood of developing diabetes [1] and various cardiovascular diseases, which can have fatal or non-fatal outcomes [2,3]. The predominant risk factors include elevated blood pressure, obesity, impaired glucose tolerance, and high cholesterol levels [4]. The World Health Organization (WHO) officially recognized the condition in 1998 and designated it as “MS.” Earlier, this condition was known as Reaven’s disease, Syndrome X, and low insulin sensitivity [5]. The prevalence of MetS has increased globally [6]. Studies in Australia and the United States reveal that approximately 20% to 25% of individuals suffer from MetS, while in India, the prevalence among adults is reported to be 31.4% [7-9]. Multiple international expert organizations have defined clinical diagnostic criteria for MetS [10]. Obesity and insulin resistance, associated with systemic inflammation, are believed to be vital in its development [7]. However, the exact mechanism by which MetS develops remains unclear [11].

Chronic obstructive pulmonary disease (COPD) is a major cause of death and a significant global health issue [12]. In 1990, COPD was the sixth leading cause of mortality worldwide, and it is predicted that by 2020, it will become the third leading cause of death and the sixth most common cause of chronic illness [13]. Although COPD is preventable and manageable, it has significant adverse effects that extend beyond the respiratory system, worsening the overall health of those affected [9-11]. Persistent airway obstruction is a defining feature of this disease. The inflammation arises when harmful particles or fumes induce abnormal reactions in the airways, typically resulting in a progressive decrease in airflow [14], which can lead to symptoms such as chronic coughing, wheezing, and shortness of breath [5].

COPD is characterized by systemic symptoms, including dyslipidemia (abnormal blood lipid levels) [2,3], anemia (low red blood cell count), osteoporosis (reduced bone density), rheumatoid arthritis (joint inflammation), diabetes [1], hypertension (high blood pressure) [3], and issues with the coronary and peripheral arteries [5]. Although the underlying mechanisms are unknown, they are believed to be associated with increased oxidative stress and inflammation [6]. Systemic inflammation in COPD can be triggered by various factors, including tissue oxygen deprivation, excessive lung expansion, impaired skeletal muscle function, steroid administration, smoking-induced pulmonary inflammation spreading to the bloodstream, activation of inflammatory cells in an inflamed lung environment, and abnormal responses from the bone marrow [15]. People with COPD commonly have elevated levels of C-reactive protein (CRP) [8].

COPD is also linked to insulin resistance, attributed to elevated levels of interleukin-6, soluble receptor I, and lipolytic tumor necrosis factor-alpha [16]. Inflammatory mediators inhibit receptor activation, leading to chronic inflammation and reduced insulin sensitivity [11]. Insulin resistance is often linked to obesity, high cholesterol levels, and hypertension, which collectively heighten cardiovascular disease and mortality risk [17]. Surprisingly, few studies have explored this topic [8-12]. Therefore, this research aims to examine the relationship between CRP levels and lymphocyte count in individuals with COPD, regardless of their MetS status.

## Materials and methods

This cross-sectional investigation encompassed 100 COPD patients admitted to the casualty ward at the Department of Biochemistry of the Index Medical College over 18 months (February 2022 to June 2023). The Institutional Ethics Committee of Malwanchal University, under reference number MU/Research/EC/Ph.D/2021/213, approved the initiation of the investigation. Initially, 263 patients were examined, of whom the inclusion and exclusion criteria led to the selection of 124. Considering a 5% attrition rate, we elected to enroll 110 patients. The required sample size of 100 was maintained throughout the study, as anticipated (95% confidence level with a margin of error of 9.8%). We used the Global Initiative for Obstructive Lung Disease (GOLD) criteria to diagnose COPD and categorize individuals [18].

Inclusion and exclusion criteria

People over 40 diagnosed with COPD were evaluated for eligibility using the GOLD criteria and categorized into four stages. Participants had to have been free of any respiratory tract infection or exacerbation for at least four weeks before the study. For this study, participants were required to complete pulmonary function tests and report no recent exacerbations. The study had extensive exclusion criteria, encompassing various specific illnesses and scenarios. We excluded people who refused consent, as well as those with chronic respiratory problems such as asthma. Participants with severe underlying health conditions or malignancies that could impact the study were also excluded. Active pulmonary tuberculosis, recent use of systemic steroids within the preceding three months, or significant exacerbations of COPD were also exclusion factors. Additionally, we excluded those with pre-existing heart issues or any cancer diagnosed within five years before the study. We gathered information about each participant’s medical history, smoking habits, and demographic characteristics.

Measurements

We measured adiposity, height, waist circumference, and systolic and diastolic blood pressure. We assessed various biomarkers, including glucose, triglycerides, total cholesterol, high-density lipoprotein (HDL), low-density lipoprotein (LDL), fibrinogen, CRP, and leukocyte count. These measurements were performed on venous blood samples collected from each participant. The subjects also underwent pulmonary function testing. We evaluated MetS using the International Diabetes Federation’s guidelines. A waist circumference of 94 cm or more was considered significant in determining the existence of MetS in both men and women. Additionally, the analysis included an assessment of factors such as a previous diagnosis of type 2 diabetes, low levels of HDL cholesterol (less than 1.03 mmol/L for men and 1.29 mmol/L for women), high levels of triglycerides (1.7 mmol/L or above), or receiving treatment for this lipid abnormality. Elevated systolic hypertension (130 mmHg or higher), elevated diastolic hypertension (85 mmHg or higher), or the presence of any 2/4 components were also considered [19].

Assessment of anthropometric variables

Weight was divided by height squared (kg/m²) to calculate the body mass index (BMI). Participants were evaluated for their weight and height while wearing non-tight-fitting clothing and without footwear. We measured waist circumference by placing a tape measure halfway between the lowest rib and the iliac crest.

Blood pressure measurements

We used a sphygmomanometer (Omron MX3 Positive, Omron, Japan) to monitor blood pressure, following American Heart Association guidelines [20].

Blood sampling and analysis

After 12 hours of fasting, a venous blood sample was obtained from each patient. We employed the hexokinase/glucose-6-phosphate dehydrogenase method to determine glucose levels. HDL cholesterol levels were determined using the direct homogeneous test, and triglyceride and total cholesterol levels were assessed using enzymatic procedures. The measurements were conducted using the Beckman-Coulter analyzer manufactured in the United States. We utilized the Friedewald equation to determine LDL cholesterol levels. CRP levels were measured using an immunoturbidimetric method with a Beckman Coulter analyzer. Fibrinogen was assessed using a clotting-based evaluation method with the BCS XP clotting analyzer produced by Siemens Healthcare Diagnostics in Germany. The leukocyte count was measured using the Beckman Coulter LH 750 hematology analyzer manufactured in the United States.

Pulmonary function testing

We performed spirometry tests using a Master Screen Pneumo spirometer made in Germany by Viasys Healthcare. Pulmonary function tests were conducted according to European Respiratory Society guidelines [16]. The measurements included forced expiratory volume in the first second (FEV1), forced vital capacity (FVC), and the ratio of FEV1 to FVC. The GOLD criteria were employed to determine the severity of COPD based on post-bronchodilator FEV1 [19].

Statistical analysis

Statistical analysis was performed using SPSS version 20 (IBM Corp., Armonk, NY, USA). We used the t-test for independent samples to compare group means and performed a chi-square test to evaluate relationships between variables. Statistical significance was defined as a p-value below 0.05.

## Results

Table 1 compares anthropometric and metabolic variables between MetS and non-MetS patients with COPD. No significant differences were observed in most parameters between patients with MetS and COPD (n = 44) and those with COPD without MetS (n = 56). The average age was 58.4 ± 8.9 years in the MetS group and 57.2 ± 9.4 years in the non-MetS group (p = 0.5181). The mean height was 166 ± 23.7 cm in the MetS group and 165 ± 26.8 cm in the non-MetS group (p = 0.84), while the mean weight was 69.7 ± 12.7 kg and 65 ± 23.2 kg, respectively (p = 0.228). The BMI was 26.3 ± 9.6 kg/m² in the MetS group versus 24 ± 9.5 kg/m² in the non-MetS group (p = 0.23). Waist circumference (96.1 ± 28.2 cm vs. 88 ± 32.1 cm) and hip circumference (99.3 ± 12.7 cm vs. 94 ± 12.8 cm) were higher in the MetS group, but these differences were not statistically significant (p = 0.18 and p = 0.213, respectively). Blood pressure readings, including systolic (138.4 ± 16.1 mmHg vs. 134.3 ± 13.2 mmHg, p = 0.164) and diastolic (91 ± 10.2 mmHg vs. 92.8 ± 9.4 mmHg, p = 0.362), did not differ significantly between the groups. However, the total white blood cell count was considerably higher in the MetS group (9,214 ± 3,161.8 cells/µL) compared to the non-MetS group (6,657.8 ± 4,218 cells/µL, p = 0.001).

**Table 1:** Comparison of anthropometric and metabolic variables between MetS and non-MetS patients with COPD. BMI: body mass index; HC: height circumference; WC: waist circumference; SBP: systolic blood pressure; DBP: diastolic blood pressure; WBC: total white blood cells; COPD: chronic obstructive pulmonary disease; MetS: metabolic syndrome

Parameters	MetS patients with COPD (n = 44)	Non-MetS patients with COPD (n = 56)	t-test value	P-value
Age (years)	58.4 ± 8.9	57.2 ± 9.4	0.6486	0.5181
Height (cm)	166 ± 23.7	165 ± 26.8	0.1948	0.84
Weight (kg)	69.7± 12.7	65 ± 23.2	1.208	0.228
BMI (kg/m²)	26.3 ± 9.6	24 ± 9.5	1.1962	0.23
WC (cm)	96.1 ± 28.2	88 ± 32.1	1.320	0.18
HC (cm)	99.3 ± 12.7	94 ± 12.8	1.253	0.213
SBP (mmHg)	138.4 ± 16.1	134.3 ± 13.2	1.399	0.164
DBP (mmHg)	91 ± 10.2	92.8 ± 9.4	0.915	0.362
Total WBC (cells/mm^3^)	9,214 ± 3,161.8	6,657.8 ± 4,218	3.348	0.001

Our results found no significant differences in smoking or hypertension between those with MetS and those without. In the MetS group (N = 44), 28 (63.6%) were smokers, while in the non-MetS group (N = 56), 42 (75%) were smokers; this difference was not statistically significant (χ² = 0.881, p = 0.347). Similarly, 25 out of 44 MetS patients (56.8%) had hypertension compared to 27 out of 56 non-MetS patients (48.2%), with no significant difference observed (χ² = 0.422, p = 0.392). However, CRP levels differed significantly; 40 (90.9%) MetS patients had elevated levels (>12 mg/L), whereas only 12 (21.4%) non-MetS patients exhibited high CRP levels (Table 2).

**Table 2:** Association of smoking, hypertension, and C-reactive protein in the study population. COPD: chronic obstructive pulmonary disease; MetS: metabolic syndrome

Variables	MetS patients with COPD (N = 44)	Non-MetS patients with COPD (N = 56)	Chi-square	P-value
Smoking			0.881	0.347
Yes	28 (63.6%)	42 (75%)		
No	16 (36.3%)	14 (25%)		
Hypertension			0.422	0.392
Yes	25 (56.8%)	27 (48.2%)		
No	19 (43.1%)	29 (51.7%)		
C-reactive protein			0.422	0.392
Positive >12 mg/L	40 (90.9%)	12 (21.4%)		
Negative <12 mg/L	4 (9.09%)	44 (78.5%)		

In our study, pulmonary function tests between patients with chronic COPD and MetS and those without MetS revealed no significant differences. The pre-bronchodilator pre-FEV1 was 1.90 ± 0.80 L in the MetS group and 1.89 ± 0.69 L in the non-MetS group (p = 0.946). Similarly, the pre-bronchodilator FEV1/FVC ratio was 0.61 ± 0.31 in the MetS group compared to 0.64 ± 0.29 in the non-MetS group (p = 0.619). Post-bronchodilator measurements showed similar trends, with post-FEV1 at 1.92 ± 0.61 L in the MetS group and 1.91 ± 0.66 L in the non-MetS group (p = 0.938). The post-bronchodilator FEV1/FVC ratio was 0.61 ± 0.22 in the MetS group versus 0.63 ± 0.19 in the non-MetS group (p = 0.627). All differences were statistically insignificant (Table 3).

**Table 3:** Comparison between COPD patients with and without MetS according to pulmonary function test values. FEV1: forced expiratory volume in one second; FEV1/FVC: forced expiratory volume in one second/forced vital capacity; COPD: chronic obstructive pulmonary disease; MetS: metabolic syndrome

Variable	MetS with COPD (n = 44)	Non-MetS with COPD (n = 56)	t-test value	P-value
Pre-FEV1(L)	1.90 ± 0.8	1.89 ± 0.69	0.067	0.946
Pre-FEV1/FVC	0.61 ± 0.31	0.64 ± 0.29	0.498	0.619
Post-FEV1(L)	1.92 ± 0.61	1.91 ± 0.66	0.077	0.938
Post-FEV1/FVC	0.61 ± 0.22	0.63 ± 0.19	0.487	0.627

## Discussion

This study examines and contrasts the demographic, clinical, and inflammatory features of individuals diagnosed with COPD who also have MetS compared with those without MetS. The study aims to enhance the understanding of these attributes and their impact on disease management. Our findings corroborate existing research while offering new insights into the intricate relationship between COPD and MetS. Data from our study confirmed earlier findings [11-17], indicating a significantly higher prevalence of COPD among male patients. This disparity is likely attributed to the higher prevalence of smoking among males, a key risk factor for COPD [21]. Our cohort included a considerable number of smokers diagnosed with COPD. The investigation revealed that 56% of smokers exhibited MetS. However, statistical analysis did not show a significant link between smoking and MetS, as detailed in Table 3. This highlights the complex nature of COPD, in which both tobacco use and metabolic factors exacerbate disease severity [22].

Individuals with COPD and MetS exhibited a higher BMI and waist circumference compared with those without MetS, based on anthropometric measurements. These findings align with previous research, which underscores the role of obesity and central adiposity in the development of COPD by impairing lung function and inducing systemic inflammation [23]. Clinical characteristics showed a slight elevation in SBP among patients with MetS and COPD. However, as shown in Table 2, this difference was not statistically significant. Our study indicates that 52% of COPD patients had hypertension, while 57% had MetS. No significant difference in MetS prevalence was observed between COPD patients with and without hypertension (p > 0.05). Consistent with our results, Patel [24] and Ghatas et al. [25] reported that 70.4% of hypertensive individuals also had COPD. Significant variations in white cell counts were observed between the two groups in our study: the first group had an average cell count of 9,214.5 cells/mm^3^. In contrast, the second group averaged 6,657.43 cells/mm^3^. Previous research has identified a correlation between MetS and severe, persistent COPD, with a notable increase in white blood cell count [26]. Regular assessment of inflammatory markers is crucial for COPD patients, particularly those with metabolic comorbidities [27]. Our study found that 40% of COPD patients had MetS, while 52% had elevated levels of CRP. MetS was observed in 4% of COPD patients with elevated CRP levels. We observed significant differences between individuals with elevated CRP levels and those with normal CRP levels. Additionally, 48% of COPD patients tested negative for CRP. Our findings align with previous research showing that 54% of COPD patients have elevated CRP levels, and 84% also have MetS [24]. Pulmonary function tests revealed no significant changes in FEV1 or FEV1/FVC ratios between groups with and without MetS, indicating similar respiratory difficulties. Prior studies on the effects of MetS on pulmonary function have yielded inconsistent results, emphasizing the need for further research to explore the underlying mechanisms and provide a comprehensive understanding of these interactions [28,29].

Limitations of the study

This study has several limitations. Precise selection of medications is crucial due to potential adverse effects on metabolic indicators; however, specific medications used by subjects were not considered. Additionally, the study did not address monitoring of glucose and lipid profiles in the sample. It is essential to consistently evaluate these indicators in COPD patients, particularly those with MetS, to optimize their condition.

## Conclusions

Our study reveals a significant association between MetS and COPD, particularly in stages two and three. COPD patients with MetS exhibited higher levels of systemic inflammation, evidenced by elevated CRP and white blood cell counts. Notably, the risk of MetS was similar for both smokers and non-smokers with COPD, highlighting the broad relevance of this comorbidity. Hypertension was common among COPD patients, reflecting shared underlying mechanisms of oxidative stress and inflammation. Importantly, pulmonary function tests showed no significant differences between COPD patients with and without MetS, suggesting that MetS primarily contributes to systemic health challenges rather than directly worsening lung function. We recommend routine screening for MetS in COPD patients, especially those in intermediate stages, to improve overall patient management and outcomes.

## References

[1] Wilson PW, D'Agostino RB, Parise H, Sullivan L, Meigs JB (2005). Metabolic syndrome as a precursor of cardiovascular disease and type 2 diabetes mellitus. Circulation.

[2] Isomaa B, Almgren P, Tuomi T (2001). Cardiovascular morbidity and mortality associated with the metabolic syndrome. Diabetes Care.

[3] Lakka HM, Laaksonen DE, Lakka TA, Niskanen LK, Kumpusalo E, Tuomilehto J, Salonen JT (2002). The metabolic syndrome and total and cardiovascular disease mortality in middle-aged men. JAMA.

[4] Acharyya A, Shahjahan MD, Mesbah FB, Dey SK, Ali L (2016). Association of metabolic syndrome with chronic obstructive pulmonary disease in an Indian population. Lung India.

[5] Kaur J (2014). A comprehensive review on metabolic syndrome. Cardiol Res Pract.

[6] Gotto AM Jr, Blackburn GL, Dailey GE 3rd, Garber AJ, Grundy SM, Sobel BE, Weir MR (2006). The metabolic syndrome: a call to action. Coron Artery Dis.

[7] Dunstan DW, Zimmet PZ, Welborn TA (2002). The rising prevalence of diabetes and impaired glucose tolerance: the Australian Diabetes, Obesity and Lifestyle Study. Diabetes Care.

[8] Ford ES, Giles WH, Dietz WH (2002). Prevalence of the metabolic syndrome among US adults: findings from the third National Health and Nutrition Examination Survey. JAMA.

[9] Das M, Pal S, Ghosh A (2011). Prevalence of cardiovascular disease risk factors by habitat: a study on adult Asian Indians in West Bengal, India. Anthropol Anz.

[10] (2001). Executive Summary of The Third Report of The National Cholesterol Education Program (NCEP) Expert Panel on Detection, Evaluation, And Treatment of High Blood Cholesterol In Adults (Adult Treatment Panel III). JAMA.

[11] Grimble RF (2002). Inflammatory status and insulin resistance. Curr Opin Clin Nutr Metab Care.

[12] Hurd S (2000). The impact of COPD on lung health worldwide: epidemiology and incidence. Chest.

[13] Lopez AD, Shibuya K, Rao C (2006). Chronic obstructive pulmonary disease: current burden and future projections. Eur Respir J.

[14] Rabe KF, Hurd S, Anzueto A (2007). Global strategy for the diagnosis, management, and prevention of chronic obstructive pulmonary disease: GOLD executive summary. Am J Respir Crit Care Med.

[15] Fabbri LM, Rabe KF (2007). From COPD to chronic systemic inflammatory syndrome?. Lancet.

[16] Bolton CE, Evans M, Ionescu AA (2007). Insulin resistance and inflammation - a further systemic complication of COPD. COPD.

[17] Després JP, Lemieux I (2006). Abdominal obesity and metabolic syndrome. Nature.

[18] (2023). Global Initiative for Chronic Obstructive Lung Disease (GOLD). Global strategy for the diagnosis, management, and prevention of COPD. http://www.goldcopd.com.

[19] Alberti KG, Zimmet P, Shaw J (2006). Metabolic syndrome--a new world-wide definition. A Consensus Statement from the International Diabetes Federation. Diabet Med.

[20] Pickering TG, Hall JE, Appel LJ (2005). Recommendations for blood pressure measurement in humans and experimental animals: Part 1: blood pressure measurement in humans: a statement for professionals from the Subcommittee of Professional and Public Education of the American Heart Association Council on High Blood Pressure Research. Hypertension.

[21] Chalmers JD, Djamin RS, Schouten M (2018). Circulating desmosine as a biomarker of azithromycin treatment response: a post hoc analysis of the COLUMBUS randomised controlled trial. ERJ Open Res.

[22] Garcia-Aymerich J, Varraso R, Danaei G (2021). Respiratory and metabolic effects of obesity in COPD patients. Respir Med.

[23] Lahousse L, Verlinden VJ, van der Geest JN (2015). Gait patterns in COPD: the Rotterdam Study. Eur Respir J.

[24] Patel B, Kadam M, Patel D, Jain U, Goyal S (2023). Obesity and COPD severity: an observational cross-sectional study. J Coastal Life Med.

[25] Ghatas T (2017). The relationship between metabolic syndrome and chronic obstructive pulmonary disease. Egypt J Bronchol.

[26] Gan WQ, Man SF, Senthilselvan A, Sin DD (2004). Association between chronic obstructive pulmonary disease and systemic inflammation: a systematic review and a meta-analysis. Thorax.

[27] Hoy R, Burdon J, Chen L (2020). Work-related asthma: a position paper from the Thoracic Society of Australia and New Zealand and the National Asthma Council Australia. Respirology.

[28] Vanfleteren LE, Spruit MA, Groenen M (2013). Clusters of comorbidities based on validated objective measurements and systemic inflammation in patients with chronic obstructive pulmonary disease. Am J Respir Crit Care Med.

[29] Casanova C, Celli BR, Barria P (2018). The obesity paradox in chronic obstructive pulmonary disease: does body mass index matter?. COPD.

